# 5-[1-(3,4-Dichloro­phen­oxy)eth­yl]-1,3,4-oxadiazole-2(*3H*)-thione hemihydrate

**DOI:** 10.1107/S1600536809029894

**Published:** 2009-08-08

**Authors:** Tashfeen Akhtar, M. Khawar Rauf, Shahid Hameed, Xiaoming Lu

**Affiliations:** a Department of Chemistry, Quaid-i-Azam University, Islamabad 45320, Pakistan; b Department of Chemistry, Capital Normal University, Beijing Taiyuan 100037, People’s Republic of China

## Abstract

In the title compound, C_10_H_8_Cl_2_N_2_O_2_S·0.5H_2_O, the atoms in the oxadiazole ring are essentially coplanar (r.m.s. deviation 0.010 Å). The crystal structure is stabilized by inter­molecular N—H⋯O hydrogen bonds involving the water mol­ecule, which is situated on an a twofold rotation axis, and two organic mol­ecules, leading to a thione tautomer in the solid state. The C atom attached to the oxadiazole ring adopts a typical *sp^3^* hybridization. The dihedral angle between the mean plane of the benzene ring of the dichloro­phenyl group and the mean plane of the oxadiazole ring is 74.18 (4)°. The crystal structure is stabilized by intermolecular N—H⋯O and O—H⋯S hydrogen bonds.

## Related literature

For the structures and properties of oxadiazoles, see: Almasirad *et al.*, (2004 ▶); Aboraia *et al.* (2006 ▶); Akhtar, Hameed, Al-Masoudi *et al.* (2008 ▶); Khan *et al.* (2005 ▶); Akhtar, Hameed *et al.* (2007 ▶); Akhtar, Hameed, Khan *et al.* (2008 ▶); Akhtar, Rauf *et al.* (2007 ▶); Aydogan *et al.*, 2002 ▶). For a related structure, see: Thamotharan *et al.* (2005 ▶). For bond-length data, see: Allen *et al.* (1987 ▶).
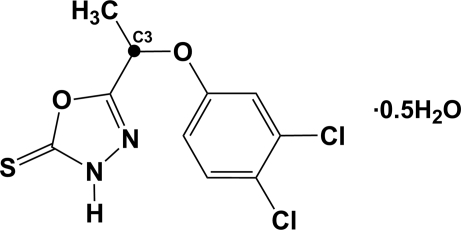


## Experimental

### 

#### Crystal data

C_10_H_8_Cl_2_N_2_O_2_S·0.5H_2_O
*M* *_r_* = 300.15
Monoclinic, 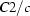

*a* = 11.8725 (2) Å
*b* = 7.89320 (10) Å
*c* = 26.6092 (4) Å
β = 92.9130 (10)°
*V* = 2490.38 (6) Å^3^
*Z* = 8
Mo *K*α radiation
μ = 0.68 mm^−1^
*T* = 123 K
0.40 × 0.30 × 0.25 mm

#### Data collection

Bruker SMART CCD area-detector diffractometer
Absorption correction: none
10568 measured reflections
3088 independent reflections
2490 reflections with *I* > 2σ(*I*)
*R*_int_ = 0.019

#### Refinement

*R*[*F* ^2^ > 2σ(*F* ^2^)] = 0.027
*wR*(*F* ^2^) = 0.078
*S* = 1.09
3088 reflections
162 parameters
H atoms treated by a mixture of independent and constrained refinement
Δρ_max_ = 0.22 e Å^−3^
Δρ_min_ = −0.21 e Å^−3^

### 

Data collection: *SMART* (Bruker, 2007 ▶); cell refinement: *SAINT* (Bruker, 2007 ▶); data reduction: *SAINT*; program(s) used to solve structure: *SHELXS97* (Sheldrick, 2008 ▶); program(s) used to refine structure: *SHELXL97* (Sheldrick, 2008 ▶); molecular graphics: *ORTEP-3* (Farrugia, 1997 ▶) and *Mercury* (Macrae *et al.*, 2006 ▶); software used to prepare material for publication: *SHELXTL* (Sheldrick, 2008 ▶).

## Supplementary Material

Crystal structure: contains datablocks I, global. DOI: 10.1107/S1600536809029894/su2129sup1.cif

Structure factors: contains datablocks I. DOI: 10.1107/S1600536809029894/su2129Isup2.hkl

Additional supplementary materials:  crystallographic information; 3D view; checkCIF report

## Figures and Tables

**Table 1:** Hydrogen-bond geometry (Å, °)

*D*—H⋯*A*	*D*—H	H⋯*A*	*D*⋯*A*	*D*—H⋯*A*
N1—H1⋯O1*W*	0.86	2.06	2.9084 (16)	168
O1*W*—H1*W*⋯S1^i^	0.846 (17)	2.612 (18)	3.3854 (13)	152.5 (17)

Symmetry code: (i) 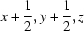.

**Fig. 1.**
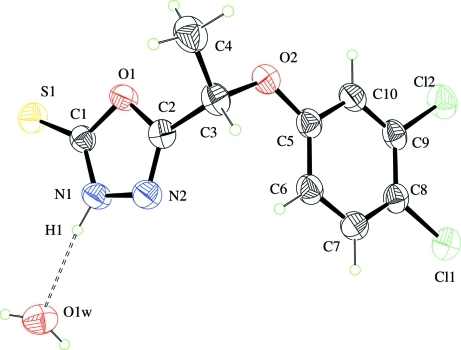
Molecular structure of the title compound, showing the atom-labelling scheme and displacement ellipsoids drawn at the 50% probability level.

**Fig. 2.**
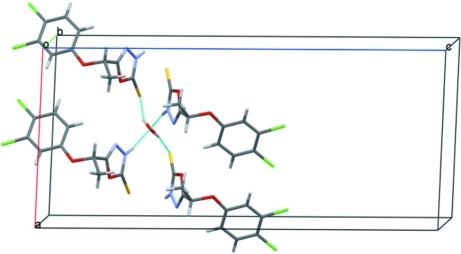
Partial crystal packing diagram of the title compound, viewed along the a axis. Hydrogen bonds are shown as dashed pale-blue lines.

## supplementary crystallographic information

### Comment

1,3,4-Oxadiazoles are an important class of five-membered heterocycles. They show diverse biological activities, for example, antifungal, antibacterial (Almasirad *et al.*, 2004), anti-convulsant (Aboraia *et al.* 2006), antitumour (Akhtar, Hameed, Khan *et al.*, 2008) and enzyme inhibitory activities (Khan *et al.*, 2005). In continuation to our work on five membered heterocycles (Akhtar & Hameed *et al.*, 2007), the title compound was synthesized and evaluated for its biological activities (Akhtar *et al.*, 2008*a*). Herein, we report on the crystal structure of 5-[1-(3,4-dichlorophenoxy) ethyl]-1,3,4-oxadiazole-2(*3H*)-thione, derived from 3,4-dichlorophenoxy propionic acid (Akhtar & Rauf *et al.*, 2007).

The molecular structure of the title compound is illustrated in Fig. 1. The dihedral angle between the mean planes of the benzene and 1,3,4-oxadiazole rings is 74.18 (4)°. The bond lengths and angles are in good agreement with the expected values (Allen *et al.*, 1987; Thamotharan *et al.*, 2005). The N1—N2 [1.3817 (16) A°] and C1=S1 [1.6489 (13) A°] bond lengths correspond to the usual single bond N—N distance and C=S distance.

1,3,4-Oxadizole-2-thiones/thiols can exist in two tautomeric forms (Aydogan *et al.*, 2002). In the title compound the thione form is observed. The H-atom of the thiol group has been transferred to the adjacent N atom of the oxadiazole ring (Fig. 1).

The title compound crystallized as a hemihydrate, with the water molecule lieing on a crystallographic two-fold axis. The water molecule O-atom hydrogen bonds to the NH group, while the water H-atom hydrogen bonds to the S-atom of the *S*=C moiety (see Table 1 and Fig. 2).

### Experimental

3,4-dichlorophenoxy acid hydrazide (10.30 mmol) was stirred with KOH (12.37 mmol) dissolved in methanol (30 ml). Carbon disulfide (0.75 mL, 12.37 mmol) was added slowly with stirring. The yellow solution obtained was refluxed until the evolution of hydrogen sulfide had ceased (18h). The reaction mixture was then cooled to rt and filtered. The filtrate was then poured into ice cooled water and acidified with 6M HCl until the colour turned congo red. The precipitate that formed was filtered off and dried. Recrystallization from ethanol/water (1:1) afforded colorless block-like crystals, suitable for X-ray diffraction analysis.

### Refinement

The water H-atom was located from a difference electron-density map and refined (O-H = 0.846 (17) Å), with *U*_iso_(H) = 1.3*U*_eq_(O). The other H-atoms were placed in idealized positions and treated as riding atoms: N-H = 0.86 Å, C—H = 0.93 - 0.98 Å with *U*_iso_(H) = 1.2*U*_eq_(N,C) or 1.5*U*_eq_(C_methyl_).

### Crystal data

**Table:**

C_10_H_8_Cl_2_N_2_O_2_S·0.5H_2_O	*F*(000) = 1224
*M**_r_* = 300.15	*D*_x_ = 1.601 Mg m^−^^3^
Monoclinic, *C*2/*c*	Mo *K*α radiation, λ = 0.71073 Å
Hall symbol: -C 2yc	Cell parameters from 4427 reflections
*a* = 11.8725 (2) Å	θ = 3.1–28.3°
*b* = 7.8932 (1) Å	µ = 0.68 mm^−^^1^
*c* = 26.6092 (4) Å	*T* = 123 K
β = 92.913 (1)°	Block, colorless
*V* = 2490.38 (6) Å^3^	0.40 × 0.30 × 0.25 mm
*Z* = 8	

### Data collection

**Table:**

Bruker SMART CCD area-detector diffractometer	2490 reflections with *I* > 2σ(*I*)
Radiation source: fine-focus sealed tube	*R*_int_ = 0.019
graphite	θ_max_ = 28.3°, θ_min_ = 3.1°
φ and ω scans	*h* = −15→15
10568 measured reflections	*k* = −10→10
3088 independent reflections	*l* = −35→35

### Refinement

**Table:**

Refinement on *F*^2^	Primary atom site location: structure-invariant direct methods
Least-squares matrix: full	Secondary atom site location: difference Fourier map
*R*[*F*^2^ > 2σ(*F*^2^)] = 0.027	Hydrogen site location: inferred from neighbouring sites
*wR*(*F*^2^) = 0.078	H atoms treated by a mixture of independent and constrained refinement
*S* = 1.09	*w* = 1/[σ^2^(*F*_o_^2^) + (0.0399*P*)^2^ + 0.7008*P*] where *P* = (*F*_o_^2^ + 2*F*_c_^2^)/3
3088 reflections	(Δ/σ)_max_ = 0.001
162 parameters	Δρ_max_ = 0.22 e Å^−^^3^
0 restraints	Δρ_min_ = −0.21 e Å^−^^3^

### Special details

**Table:**

Geometry. All e.s.d.'s (except the e.s.d. in the dihedral angle between two l.s. planes) are estimated using the full covariance matrix. The cell e.s.d.'s are taken into account individually in the estimation of e.s.d.'s in distances, angles and torsion angles; correlations between e.s.d.'s in cell parameters are only used when they are defined by crystal symmetry. An approximate (isotropic) treatment of cell e.s.d.'s is used for estimating e.s.d.'s involving l.s. planes.
Refinement. Refinement of *F*^2^ against ALL reflections. The weighted *R*-factor *wR* and goodness of fit *S* are based on *F*^2^, conventional *R*-factors *R* are based on *F*, with *F* set to zero for negative *F*^2^. The threshold expression of *F*^2^ > σ(*F*^2^) is used only for calculating *R*-factors(gt) *etc*. and is not relevant to the choice of reflections for refinement. *R*-factors based on *F*^2^ are statistically about twice as large as those based on *F*, and *R*- factors based on ALL data will be even larger.

### Fractional atomic coordinates and isotropic or equivalent isotropic displacement parameters (Å^2^)

**Table:**

	*x*	*y*	*z*	*U*_iso_*/*U*_eq_	
O1	0.25873 (7)	0.32348 (12)	0.33123 (4)	0.0347 (2)	
C1	0.26630 (11)	0.47444 (17)	0.30643 (5)	0.0321 (3)	
S1	0.15653 (3)	0.59419 (5)	0.290316 (15)	0.04313 (11)	
N1	0.37606 (9)	0.49544 (15)	0.30057 (4)	0.0373 (3)	
H1	0.4040	0.5814	0.2857	0.045*	
N2	0.44029 (9)	0.36407 (16)	0.32095 (5)	0.0379 (3)	
C2	0.36727 (11)	0.26539 (17)	0.33892 (5)	0.0330 (3)	
C3	0.38524 (12)	0.09920 (18)	0.36521 (5)	0.0381 (3)	
H3	0.4630	0.0612	0.3616	0.046*	
C4	0.30442 (15)	−0.0347 (2)	0.34486 (6)	0.0495 (4)	
H4A	0.3187	−0.1393	0.3625	0.074*	
H4B	0.3148	−0.0510	0.3096	0.074*	
H4C	0.2284	0.0010	0.3495	0.074*	
O2	0.36403 (8)	0.11531 (14)	0.41765 (4)	0.0417 (2)	
C5	0.44340 (11)	0.18810 (17)	0.45029 (5)	0.0351 (3)	
C6	0.54468 (11)	0.25923 (18)	0.43709 (5)	0.0374 (3)	
H6	0.5649	0.2584	0.4038	0.045*	
C7	0.61526 (11)	0.33147 (19)	0.47429 (5)	0.0379 (3)	
H7	0.6830	0.3797	0.4656	0.046*	
C8	0.58683 (11)	0.33307 (17)	0.52386 (5)	0.0354 (3)	
Cl1	0.67766 (3)	0.42681 (5)	0.568937 (14)	0.04480 (11)	
C9	0.48523 (12)	0.26045 (17)	0.53664 (5)	0.0360 (3)	
Cl2	0.44427 (3)	0.26092 (6)	0.598122 (14)	0.05328 (13)	
C10	0.41405 (11)	0.18835 (18)	0.50013 (5)	0.0380 (3)	
H10	0.3464	0.1399	0.5089	0.046*	
O1W	0.5000	0.75760 (19)	0.2500	0.0457 (4)	
H1W	0.5445 (15)	0.817 (2)	0.2687 (7)	0.059*	

### Atomic displacement parameters (Å^2^)

**Table:**

	*U*^11^	*U*^22^	*U*^33^	*U*^12^	*U*^13^	*U*^23^
O1	0.0320 (4)	0.0336 (5)	0.0389 (5)	0.0003 (4)	0.0056 (4)	0.0056 (4)
C1	0.0360 (6)	0.0313 (7)	0.0291 (6)	0.0000 (5)	0.0026 (5)	−0.0009 (5)
S1	0.03808 (18)	0.0406 (2)	0.0504 (2)	0.00707 (14)	−0.00080 (15)	0.00403 (16)
N1	0.0354 (5)	0.0342 (6)	0.0425 (6)	0.0001 (5)	0.0062 (5)	0.0078 (5)
N2	0.0337 (5)	0.0384 (6)	0.0419 (6)	0.0037 (5)	0.0046 (5)	0.0045 (5)
C2	0.0349 (6)	0.0327 (7)	0.0315 (6)	0.0034 (5)	0.0032 (5)	−0.0013 (5)
C3	0.0434 (7)	0.0358 (7)	0.0355 (7)	0.0051 (6)	0.0045 (6)	0.0038 (6)
C4	0.0664 (10)	0.0345 (8)	0.0473 (9)	−0.0019 (7)	−0.0005 (7)	0.0028 (7)
O2	0.0414 (5)	0.0494 (6)	0.0344 (5)	−0.0037 (4)	0.0027 (4)	0.0062 (4)
C5	0.0356 (6)	0.0341 (7)	0.0356 (7)	0.0075 (5)	0.0025 (5)	0.0065 (6)
C6	0.0361 (7)	0.0437 (8)	0.0328 (7)	0.0066 (6)	0.0058 (5)	0.0059 (6)
C7	0.0335 (6)	0.0409 (8)	0.0400 (7)	0.0065 (6)	0.0065 (5)	0.0052 (6)
C8	0.0361 (6)	0.0332 (7)	0.0371 (7)	0.0098 (5)	0.0019 (5)	0.0023 (6)
Cl1	0.04519 (19)	0.0466 (2)	0.0423 (2)	0.00724 (15)	−0.00057 (14)	−0.00463 (16)
C9	0.0402 (7)	0.0363 (7)	0.0321 (7)	0.0119 (6)	0.0074 (5)	0.0071 (6)
Cl2	0.0564 (2)	0.0696 (3)	0.03508 (19)	0.00834 (19)	0.01402 (16)	0.00641 (18)
C10	0.0364 (7)	0.0380 (8)	0.0401 (7)	0.0051 (6)	0.0078 (6)	0.0096 (6)
O1W	0.0431 (8)	0.0329 (8)	0.0612 (10)	0.000	0.0048 (7)	0.000

### Geometric parameters (Å, °)

**Table:**

O1—C1	1.3672 (16)	O2—C5	1.3745 (17)
O1—C2	1.3733 (15)	C5—C10	1.3882 (19)
C1—N1	1.3306 (16)	C5—C6	1.3884 (19)
C1—S1	1.6489 (13)	C6—C7	1.387 (2)
N1—N2	1.3817 (16)	C6—H6	0.9300
N1—H1	0.8600	C7—C8	1.3783 (19)
N2—C2	1.2763 (18)	C7—H7	0.9300
C2—C3	1.4967 (19)	C8—C9	1.3931 (19)
C3—O2	1.4363 (17)	C8—Cl1	1.7371 (15)
C3—C4	1.509 (2)	C9—C10	1.378 (2)
C3—H3	0.9800	C9—Cl2	1.7302 (14)
C4—H4A	0.9600	C10—H10	0.9300
C4—H4B	0.9600	O1W—H1W	0.846 (17)
C4—H4C	0.9600		
			
C1—O1—C2	106.18 (10)	H4A—C4—H4C	109.5
N1—C1—O1	104.71 (11)	H4B—C4—H4C	109.5
N1—C1—S1	131.59 (11)	C5—O2—C3	120.17 (10)
O1—C1—S1	123.69 (9)	O2—C5—C10	114.02 (12)
C1—N1—N2	112.63 (11)	O2—C5—C6	125.73 (12)
C1—N1—H1	123.7	C10—C5—C6	120.25 (14)
N2—N1—H1	123.7	C7—C6—C5	119.04 (13)
C2—N2—N1	103.48 (10)	C7—C6—H6	120.5
N2—C2—O1	112.99 (12)	C5—C6—H6	120.5
N2—C2—C3	128.79 (12)	C8—C7—C6	121.23 (13)
O1—C2—C3	118.20 (11)	C8—C7—H7	119.4
O2—C3—C2	110.40 (11)	C6—C7—H7	119.4
O2—C3—C4	105.72 (12)	C7—C8—C9	119.15 (13)
C2—C3—C4	111.92 (12)	C7—C8—Cl1	119.41 (11)
O2—C3—H3	109.6	C9—C8—Cl1	121.44 (10)
C2—C3—H3	109.6	C10—C9—C8	120.36 (12)
C4—C3—H3	109.6	C10—C9—Cl2	118.41 (11)
C3—C4—H4A	109.5	C8—C9—Cl2	121.22 (11)
C3—C4—H4B	109.5	C9—C10—C5	119.97 (13)
H4A—C4—H4B	109.5	C9—C10—H10	120.0
C3—C4—H4C	109.5	C5—C10—H10	120.0

### Hydrogen-bond geometry (Å, °)

**Table:**

*D*—H···*A*	*D*—H	H···*A*	*D*···*A*	*D*—H···*A*
N1—H1···O1W	0.86	2.06	2.9084 (16)	168
O1W—H1W···S1^i^	0.846 (17)	2.612 (18)	3.3854 (13)	152.5 (17)

Symmetry codes: (i) *x*+1/2, *y*+1/2, *z*.
